# Local Riemannian geometry of model manifolds and its implications for practical parameter identifiability

**DOI:** 10.1371/journal.pone.0217837

**Published:** 2019-06-03

**Authors:** Daniel Lill, Jens Timmer, Daniel Kaschek

**Affiliations:** 1 Institute of Physics, University of Freiburg, Freiburg, Germany; 2 BIOSS Centre For Biological Signalling Studies, University of Freiburg, Freiburg, Germany; Delft University of Technology, NETHERLANDS

## Abstract

When non-linear models are fitted to experimental data, parameter estimates can be poorly constrained albeit being identifiable in principle. This means that along certain paths in parameter space, the log-likelihood does not exceed a given statistical threshold but remains bounded. This situation, denoted as practical non-identifiability, can be detected by Monte Carlo sampling or by systematic scanning using the profile likelihood method. In contrast, any method based on a Taylor expansion of the log-likelihood around the optimum, e.g., parameter uncertainty estimation by the Fisher Information Matrix, reveals no information about the boundedness at all. In this work, we present a geometric approach, approximating the original log-likelihood by geodesic coordinates of the model manifold. The Christoffel Symbols in the geodesic equation are fixed to those obtained from second order model sensitivities at the optimum. Based on three exemplary non-linear models we show that the information about the log-likelihood bounds and flat parameter directions can already be contained in this local information. Whereas the unbounded case represented by the Fisher Information Matrix is embedded in the geometric framework as vanishing Christoffel Symbols, non-vanishing constant Christoffel Symbols prove to define prototype non-linear models featuring boundedness and flat parameter directions of the log-likelihood. Finally, we investigate if those models could allow to approximate and replace computationally expensive objective functions originating from non-linear models by a surrogate objective function in parameter estimation problems.

## Introduction

Parameter estimation by the maximum-likelihood method has numerous applications in different fields of physics, engineering, and other quantitative sciences. In systems biology, e.g., ordinary differential equation (ODE) models are used to describe cell-biological processes [1, 2]. Parameter estimation in these non-linear models can easily become time-consuming. Solving the ODEs and computing model sensitivities for numerical optimization is computationally demanding. The difficulty is further increased if many experimental conditions contribute to the evaluation of the likelihood function because the model ODE needs to be solved independently for each condition.

Upon successful parameter estimation, thorough investigation of the log-likelihood around the optimum frequently reveals that some parameters, although having a unique optimum, cannot be constrained to finite confidence intervals. This situation is denoted as practical non-identifiability [3]. The reason for practical non-identifiability is the non-linear relationship between model parameters and model predictions. The non-linearity culminates in the boundedness of model predictions for all possible combinations of parameters and, consequently, in upper limits of the negative log-likelihood that are not exceeded along certain paths. Based on the likelihood-ratio test statistics, log-likelihood thresholds relative to the value at the optimum can be derived [4] that, when being exceeded by the choice of parameters, allow to reject the model specification. Conversely, if the derived thresholds are above the upper limit of the negative log-likelihood, the model cannot be rejected over an infinite range of parameter values.

In this work we discuss that for certain models, practical non-identifiability can already be detected from local information, i.e., *second order model sensitivities* at the optimum. The approach even allows to construct an approximated log-likelihood function uniting both, the local shape around the optimum and the asymptotic shape in the limit of arbitrarily large/small parameter values. The construction is based on a differential geometric point of view on least squares estimation as laid out in [5, 6]. The geometry of least squares estimation has already previously been discussed, e.g., in [7]. Also the usage of second order model sensitivities to derive equations for parameter transformations providing the log-likelihood with a more quadratic shape around the optimum has been suggested in earlier statistical works, see [8, 9]. However, these previous attempts have been too general to be either solved analytically or be feasible numerically.

In contrast, by sticking to a local approximation of Christoffel Symbols, i.e., the connection coefficients of the Levi-Civita connection on the model manifold, [10], we can show that these are sufficient to construct a globally defined parameter transformation with bounded co-domain that, in the best case, turns the original log-likelihood into a purely quadratic function of the new coordinates. The boundary value problem underlying the parameter transformation can be solved efficiently by numerical methods [11]. The result is that despite being based on purely local second order sensitivity information, the log-likelihood function constructed in this way reflects a fundamental property of the original log-likelihood: its boundedness.

It is thereby possible to capture not only the parameter estimates but also their correlation structure locally as well as in the limit of practical non-identifiability.

## Methods

### The statistical point of view

Given a mathematical model to describe a set of *M* data points, one is interested in the *N* parameters such that the model fits the data best. In this work, a data point *y*_*D*_ taken at the value *t*_*m*_ (usually time) is assumed to be described by a model *y*(*t*, *θ*) evaluated at the parameter $\theta =\theta_{\text{true}}\in P\subseteq R^{N}$. Additionally, data points are affected by Gaussian noise *ϵ* ∼ *N*(0, *σ*^2^):

$$\begin{array}{c} y_{D,m}=y\left(t_{m},\theta_{\text{true}}\right)+\epsilon_{m}. \end{array}$$ (1)

In the case of Gaussian noise and known variance *σ*^2^, the Maximum Likelihood Estimate for the parameters is given by

$$\begin{array}{c} \hat{\theta }=\underset{\theta }{\mathrm{argmin}}\underset{\chi^{2}\left(\theta \right)}{\underset{︸}{\sum_{m=1}^{M}(\frac{y_{D,m}-y\left(t_{m},\theta \right)}{\sigma_{m}}{)}^{2}}}. \end{array}$$ (2)

*χ*^2^ is a non-linear function of the parameters, and therefore can be bounded in certain directions of the parameter space, in which case we call this model a bounded model. This boundedness has implications for parameter estimation and confidence interval determination [3]. The confidence interval $I_{\theta_{i}}\subseteq R$ of the $i^{\text{th}}$ parameter is defined as

$$\begin{array}{c} I_{\theta_{i}}=\left\{\theta_{i}|\underset{\theta_{j}}{\text{min}}\chi^{2}\left(\theta_{i},{\left\{\theta_{j}\right\}}_{j\neq i}\right)-\chi^{2}\left(\hat{\theta }\right)<T_{1-\alpha }\right\}, \end{array}$$ (3)

where *T*_1−*α*_ is the threshold to be exceeded to guarantee a confidence level of 1 − *α*. These confidence intervals can be either smaller or larger than those derived from the Fisher Information Matrix, or equal, in case of a purely quadratic shape of *χ*^2^. Eq (3) implies that the boundedness of *χ*^2^ eventually leads to infinite confidence intervals if the confidence level is chosen large enough.

### The geometric point of view

A different perspective on the boundedness of *χ*^2^ can be obtained by regarding it as a function of the normalized residuals *r*^*m*^. All residuals can be combined into a vector *r* which is an element of the *M*-dimensional *data space* D. In D, each residual contributes one dimension and the *χ*^2^ function is simply a quadratic function of *r*:

$$\begin{array}{c} \chi^{2}=\sum_{m}(\frac{y_{D,m}-y\left(t_{m},\theta \right)}{\sigma_{m}}{)}^{2}=\sum_{m}(r^{m}\left(\theta \right){)}^{2}=\|r(\theta ){\|}^{2} \end{array}$$ (4)

The residual vector is restricted to the *N*-dimensional model manifold M, which is the set of all residual vectors that can be reached by the model:

$$\begin{array}{c} M=\{r|r=r(\theta ),\theta \in P\}. \end{array}$$ (5)

**Fig 1:**
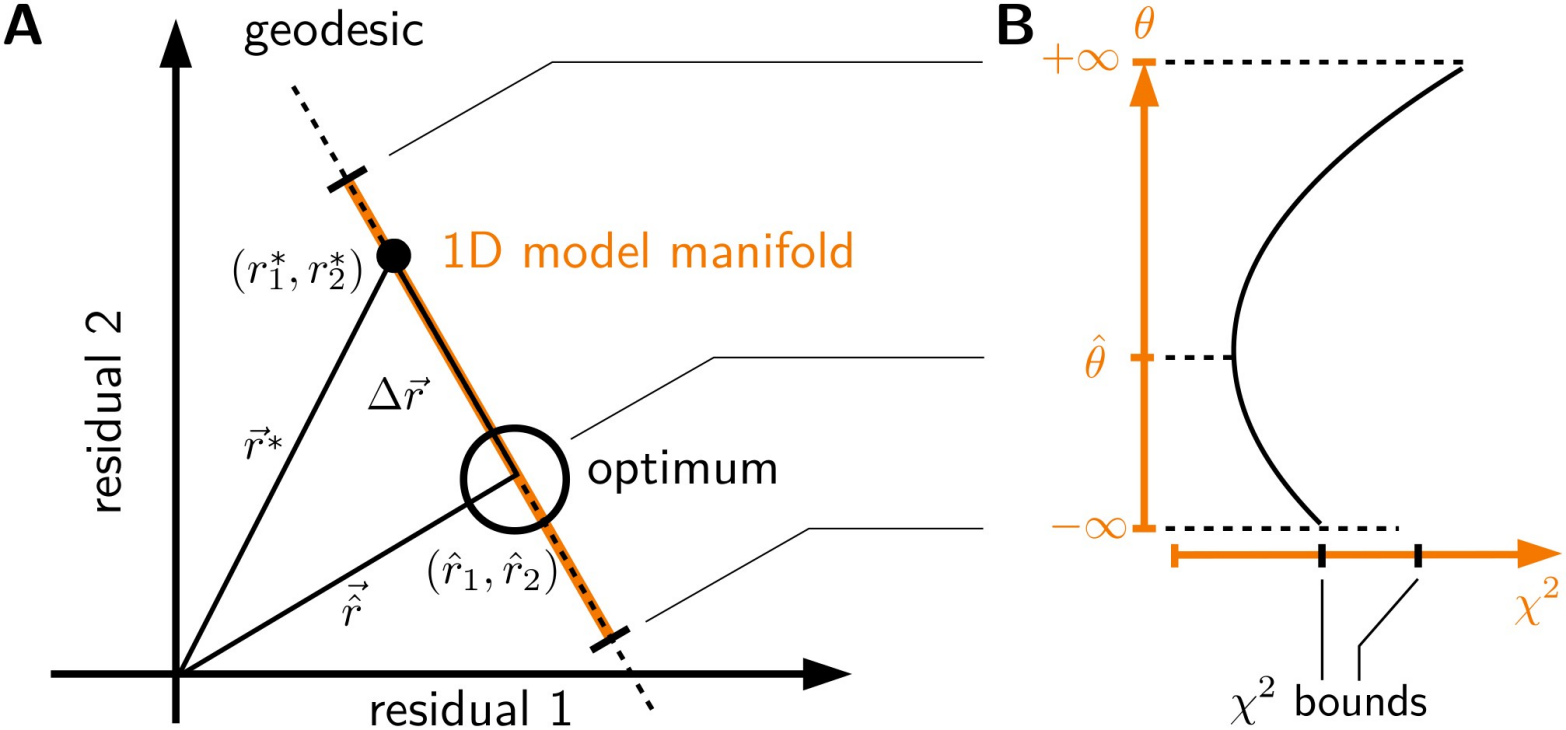
Model manifold with boundary. (A) The tangent at the optimum is perpendicular to its residual vector $\vec{\hat{r}}$. Boundaries of the model manifold, shown in orange, are marked by black segments. In the parameterization by the model parameter *θ* the boundaries are reached in the limit *θ* → ±∞. (B) The values of *χ*^2^ on the interval [−∞, ∞] are shown as graph, illustrating the boundedness of the function.

With the parameters θ∈P, M is readily equipped with a coordinate system. Fig 1A shows an example of an extrinsically flat, one-dimensional model manifold in a two-dimensional data space. Fig 1B shows *χ*^2^ in the parameter space. This simple manifold illustrates that the boundedness of the *χ*^2^ function in P is reflected in D via boundaries of the model manifold. Even tuning the parameter *θ* to infinity results in a residual vector of finite length.

At the optimum $\hat{r}$, any tangent vector of the model manifold is perpendicular to the residual vector itself, as emphasized in Fig 1A, and the squared distance between a point r* on the flat model manifold and $\hat{r}$ can be directly related to a change in *χ*^2^:

$$\begin{array}{c} \Delta \chi^{2}=\chi^{2}\left(r^{*}\right)-\chi^{2}\left(\hat{r}\right)=\|\Delta r{\|}^{2}. \end{array}$$ (6)

We now construct a coordinate system for M to take advantage of this geometric property. In the Euclidean data space, the length of the vector connecting $\hat{r}$ and r* coincides with the arc length *s* of the geodesic between these points. We parameterize this geodesic *r*(*τ*), solution to the equation $\ddot{r}=0$ by

$$\begin{array}{c} r\left(\tau \right)=\hat{r}+\frac{r^{*}-\hat{r}}{\Delta \tau }\tau =\hat{r}+v_{\hat{r}}\tau \end{array}$$ (7)

with Δ*τ* = 1. The squared arc length *s*^2^ can therefore be expressed simply in terms of the velocity $v_{\hat{r}}=\Delta r/\Delta \tau$ of the geodesic:

$$\begin{array}{c} s^{2}={\left\|\Delta r\right\|}^{2}=\int_{0}^{1}\delta_{ij}{\dot{r}}^{i}{\dot{r}}^{j}d\tau =\delta_{ij}v_{\hat{r}}^{i}v_{\hat{r}}^{j}. \end{array}$$ (8)

Here, *δ*_*ij*_ is the Euclidean metric of D, and we make use of Einstein’s summation convention. Working in the coordinate system of model parameters *θ* the geodesic equation needs to be solved for the metric of P. Compared to the Euclidean data space, these expressions take the following form:

$$\begin{array}{c} \begin{array}{cc} \delta_{ij}\;⇝\; & g_{\mu \nu }=\frac{\partial r^{m}}{\partial \theta^{\mu }}\frac{\partial r^{n}}{\partial \theta^{\nu }}\delta_{mn}\\ \ddot{r}=0\;⇝\; & {\ddot{\theta }}^{\mu }=-\Gamma_{\alpha \beta }^{\mu }{\dot{\theta }}^{\alpha }{\dot{\theta }}^{\beta }, \end{array} \end{array}$$ (9)

with non-vanishing Christoffel Symbols of the second kind, referred to as “Christoffel Symbols” in this work:

$$\begin{array}{c} \Gamma_{\alpha \beta }^{\mu }\;=\;\sum_{m}g^{\mu \nu }\frac{\partial r^{m}}{\partial \theta^{\nu }}\frac{\partial^{2}r^{m}}{\partial \theta^{\alpha }\partial \theta^{\beta }}, \end{array}$$ (10)

where *g*^*μν*^ = (*g*_*μν*_)^−1^ [6].

As a defining property of geodesics, the absolute value of the velocity stays constant along a geodesic. Thus, initial velocities of the geodesic at $\hat{\theta }$ suffice to express *s*^2^ in the coordinate system of model parameters

$$\begin{array}{c} s^{2}=\int_{0}^{1}g_{\mu \nu }{\dot{\theta }}^{\mu }{\dot{\theta }}^{\nu }d\tau =g_{\mu \nu }{|}_{\hat{\theta }}v_{\hat{\theta }}^{\mu }v_{\hat{\theta }}^{\nu } \end{array}$$ (11)

with $v_{\hat{\theta }}$ indicating that the initial velocity is now expressed in *θ*-coordinates. The initial velocities are in fact Riemann Normal Coordinates (RNC) for M [10]. For bounded model manifolds, the RNC are bounded in their domain, since the boundary can be reached by a geodesic with finite initial velocity.

Combining Eqs (6) and (11), *χ*^2^ transforms from a non-linear into a quadratic function:

$$\begin{array}{c} \chi^{2}\left(\theta \right)\;⇝\;\chi^{2}\left(v_{\hat{\theta }}\right)\approx \chi^{2}{|}_{\hat{\theta }}+g_{\mu \nu }{|}_{\hat{\theta }}v_{\hat{\theta }}^{\mu }v_{\hat{\theta }}^{\nu }. \end{array}$$ (12)

The boundedness of this expression is now achieved by the finite domain of the coordinates $v_{\hat{\theta }}$ rather than through the model’s non-linearity.

We emphasize that Eq (12) is exact if and only if the model manifold is extrinsically flat. For non-linear models, the extrinsic curvature generally is non-zero and Eq (12) only holds locally, since in this case the assumption $\Delta r⊥\hat{r}$ in Eq (6) is violated when moving further away from the optimum and the geodesic is not a straight path in D. In this work, we do not account for this deviation. It has been noted in [6] that extrinsic curvature of model manifolds can often be neglected.

## Results

The methods described above can be used to approximate *χ*^2^ in a new way to allow for regions in P where *χ*^2^ is bounded.

To perform the coordinate change from the original parameters to the RNC, the geodesic equation has to be solved as a two-point boundary value problem. The first point is the point around which the RNC are constructed, in our case $\hat{\theta }$. The second one is the point where *χ*^2^(*θ*) is to be approximated. Since the geodesic equation is a non-linear ordinary differential equation, in most cases a closed-form solution does not exist and approximations are made to solve the geodesic equation. A popular approach in literature (e.g. [6]) is to Taylor expand all objects in the geodesic equation in terms of the curve parameter *τ* and requiring that, locally, the geodesic has the form of a straight line. The coordinates *v*(*θ*) obtained this way are polynomials of *θ*. As a polynomial, the approximated expression for *χ*^2^(*θ*) cannot be bounded and hence, the accuracy of the approximation becomes insufficient for asymptotically bounded *χ*^2^. On the other hand, solving the geodesic equation numerically comes with high computational costs because at each integration step, the model’s derivatives up to second order must be computed to evaluate the Christoffel Symbols.

Our approach approximates only the Christoffel Symbols by their values at $\hat{\theta }$ and inserts them in the otherwise unmodified geodesic equation:

$$\begin{array}{c} {\ddot{\theta }}^{\mu }=-\Gamma_{\alpha \beta }^{\mu }{|}_{\theta =\hat{\theta }}{\dot{\theta }}^{\alpha }{\dot{\theta }}^{\beta }. \end{array}$$ (13)

This approximated geodesic equation can be numerically solved without repeated model evaluation. The parameter transformation between original parameters *θ* and RNC *v* is given by

$$\begin{array}{c} \begin{array}{cc} \phi :\theta^{\mu }\mapsto v^{\mu }= & \;{\dot{\theta }}^{\mu }\left(0\right)\\ & \;s.t.\theta^{\mu }\left(0\right)={\hat{\theta }}^{\mu },\;\theta^{\mu }\left(1\right)=\theta^{\mu }, \end{array} \end{array}$$ (14)

where *θ*^*μ*^(⋅) denotes the solution of Eq (13).

By construction, the resulting curves approximate the true geodesics in a neighborhood of $\hat{\theta }$. The approximated RNC *v* are inserted in Eq (12) with the metric $g_{\mu \nu }{|}_{\theta =\hat{\theta }}$ to obtain an approximation of *χ*^2^: Through the Christoffel Symbols, this approximation depends on second order model sensitivities at $\hat{\theta }$, but unlike a Taylor expansion of *χ*^2^ of order two, it allows for areas in the parameter space, in which it is bounded. This can be understood from the fact that the solution of a quadratic ordinary differential equation can diverge in finite time. In other words, infinite values for the original parameters *θ* can be obtained from finite values of the new parameters *v*. Furthermore, local skewness of *χ*^2^ around the optimum can be better captured than by the Hessian matrix.

Summarising, the approximated objective function can be implemented by the following steps:

1. Optimize the original log-likelihood *χ*^2^(*θ*) to obtain the optimal parameter vector $\hat{\theta }$.
2. Compute the first order and second order sensitivities of the residuals at the optimum. Note that derivatives of the residuals are required, as opposed to derivatives of the objective function $\frac{\partial r^{m}}{\partial \theta^{\mu }}{|}_{\theta =\hat{\theta }}$ and $\frac{\partial^{2}r^{m}}{\partial \theta^{\mu }\partial \theta^{\nu }}{|}_{\theta =\hat{\theta }}.$
3. With these sensitivities, compute the values of the metric ${\hat{g}}_{\mu \nu }=g_{\mu \nu }{|}_{\hat{\theta }}$ and the Christoffel Symbols ${\hat{\Gamma }}_{\alpha \beta }^{\mu }=\Gamma_{\alpha \beta }^{\mu }{|}_{\hat{\theta }}$ according to Eqs (9) and (10).
4. With the Christoffel Symbols $\hat{\Gamma }$, solve the geodesic equation as boundary value problem with the constraints specified by Eq (14). To this end, it usually is helpful to reformulate the geodesic equation as a first order ODE with auxiliary variables *ξ*:
  $$\begin{array}{c} \begin{array}{cc} \dot{\theta^{\mu }} & =\xi^{\mu }\\ \dot{\xi^{\mu }} & =-{\hat{\Gamma }}_{\alpha \beta }^{\mu }\xi^{\alpha }\xi^{\beta } \end{array} \end{array}$$ (15)
5. From the solution, use the initial velocities $v={\dot{\theta }}_{\tau =0}$ as new RNC to obtain the approximated ${\tilde{\chi }}^{2}$:
  $$\begin{array}{c} {\tilde{\chi }}^{2}\left(v\left(\theta \right)\right)\approx \chi^{2}{|}_{\hat{\theta }}+{\hat{g}}_{\mu \nu }v^{\mu }\left(\theta \right)v^{\nu }\left(\theta \right) \end{array}$$ (16)
  For applications relying on derivative information of the objective function, we provide formulas to obtain the gradient and an approximated Hessian of ${\tilde{\chi }}^{2}$ in section D in S1 Text.

### Model 1: Exponential growth/decay with fixed initial amount

We discuss various features of the approximation by means of three models with increasing complexity. The first model is an example for which the approximation is exact. Consider the model

$$\begin{array}{c} y=e^{-k_{1}t}, \end{array}$$ (17)

with $k_{1}\in R$. The sign of the parameter *k*_1_ determines whether the model describes exponential decay or exponential growth.

If we measure only one data point *y*_*D*_ = 1 at *t* = 1 with standard deviation *σ* = 1, the objective function is given by $\chi^{2}={\left(1-e^{-k_{1}}\right)}^{2}$, the model manifold is one-dimensional, extrinsically flat and its Christoffel Symbol is given by $\Gamma_{11}^{1}=-1$. Clearly, the model manifold is bounded in one direction: In a one-dimensional data space, it covers the positive real numbers shifted negatively by the value of the data point. In this rare case, the geodesic equation can be solved exactly (detailed steps are presented in section E in S1 Text):

$$\begin{array}{c} \theta \left(\tau \right)=-\text{log}\left(C_{1}\tau +C_{0}\right) \end{array}$$ (18)

The integration constants are chosen as

$$C_{0}=1,$$ (19)

$$C_{1}=-v$$ (20)

This way, the boundary conditions at *τ* = 0 and *τ* = 1 are fulfilled and the initial velocity is given by $\dot{\theta }{|}_{\tau =0}=v$. The coordinate change can now be performed by substitution of the solution at *τ* = 1 into the original *χ*^2^—function:

$$\begin{array}{c} \chi^{2}\left(v\right)=(e^{-k_{1}}-y_{D}{)}^{2}=(e^{-(-\log(-v+1))}-y_{D}{)}^{2}=(v{)}^{2} \end{array}$$ (21)

Since the Christoffel Symbols are constant and the model manifold is extrinsically flat, this amounts to the same expression as if Eq (16) is used directly, with $g_{\mu \nu }{|}_{\hat{\theta }}=1$:

$$\begin{array}{c} {\tilde{\chi }}^{2}\left(v\right)=\chi^{2}{|}_{\hat{\theta }}+g_{\mu \nu }{|}_{\hat{\theta }}v_{\hat{\theta }}^{\mu }v_{\hat{\theta }}^{\nu }=0+1\cdot v^{2} \end{array}$$ (22)

As previously derived, the *χ*^2^—function is transformed back to a quadratic function by the Riemann Normal Coordinates, but the domain of *v* is restricted to values smaller than 1.

The source of the boundedness of the *χ*^2^—function is clearly the restricted co-domain of the model itself. We therefore conclude that boundedness of a model along a parameter axis relates to a restricted co-domain of the model: The amount of the decaying substance can never drop below zero, regardless of the rate constant. This boundedness is represented appropriately by a model with constant Christoffel Symbol.

### Model 2: Exponential decay with variable initial amount

We now modify Model 1 at two stages: On the one hand, we introduce a parameter *A*_0_ for the initial amount:

$$\begin{array}{c} \dot{A}=-k_{1}A\;\Leftrightarrow \;A\left(t\right)=A_{0}e^{-k_{1}t}. \end{array}$$ (23)

**Fig 2:**
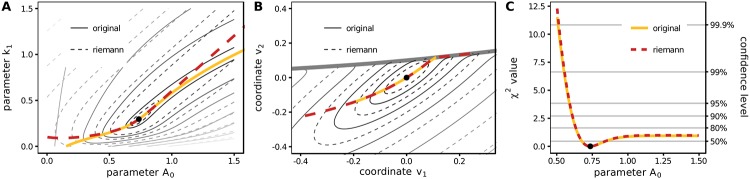
Landscape and profile of *χ*^2^. (A) The shape of the landscape is visualized by solid and dashed contour lines for the original and Riemann approximated *χ*^2^, respectively. The colored lines represent paths that are optimal with respect to the parameter *k*_1_ for any given value of parameter *A*_0_. (B) The non-quadratic *χ*^2^ turns into a quadratic function in Riemannian Normal Coordinates. The paths computed for (A) are shown in the new coordinates as colored lines. (C) The *χ*^2^ values along the exact and the approximated parameter paths agree well indicating that confidence intervals derived from either objective function coincide. Thresholds for different confidence levels are depicted in gray.

On the other hand, we restrict both the parameter *k*_1_ and *A*_0_ to values greater or equal to zero. Fig 2 visualizes an exemplary *χ*^2^ landscape for three data points the values of which are presented in section B in S1 Text. In Fig 2A, the contours of the original objective function are shown as solid line.

The restricted model behaviour as in Model 1 can again be observed when considering one-dimensional cross sections of the *χ*^2^ landscape for a fixed value of the initial amount *A*_0_. Furthermore, and somewhat trivially, the *χ*^2^ values are bounded as soon as the parameters approach their respective boundaries. However, also the third source of boundedness can be observed, the coupling of parameters such that a flat canyon is formed which means that a change of one parameter is compensated by the other parameter.

The dashed contour lines represent the approximated objective function ${\tilde{\chi }}^{2}$. In comparison to the original objective function, the asymptotic behaviour for cross sections at constant *A*_0_ does not appear to be bounded, but for cross sections at constant *k*_1_. However, the parameter coupling between *A*_0_ and *k*_1_ is matched very well, a behavior which an approximation by a Taylor expansion could never exhibit. It is noticeable that the path of the parameter coupling is straight as opposed to the curved path of the original objective function. However, we note that usually the paths of parameter coupling tend to straighten out asymptotically.

The red and yellow lines, referring to *χ*^2^ and ${\tilde{\chi }}^{2}$, respectively, indicate the paths that for given value of *A*_0_ minimize *χ*^2^ with respect to *k*_1_, the so-called profile likelihood path for parameter *A*_0_. They each follow the paths of the parameter coupling, which, though different in parameter space, appear to have very similar objective function values along their path, which is shown in Fig 2C.

The same paths and contours are shown in Fig 2B in the new coordinates *v*. By construction, the dashed contour-lines of the approximated *χ*^2^ are exactly elliptic, but with a boundary as indicated by the fat gray line. Also the original *χ*^2^ appears more quadratic in the new coordinates.

The approximated *χ*^2^ purely based on local information around the optimum correctly describes the non-linear phenomenon of parameter coupling. The identification of parameter coupling in the limit of infinitely large/small parameters is a key element of model reduction as demonstrated in [12] and [13].

### Model 3: Enzyme kinetics

Next, the approximation is tested on an enzymatic reaction modeled by mass-action kinetics. In this model, an enzyme *E* and its substrate *S* first form a complex *C* which can either dissociate back into *E* and *S*, or form a product *P*, in which case *P* and *E* are released. The corresponding ODEs are given by

$$\begin{array}{c} \begin{array}{cc} \dot{\left[S\right]} & =-k_{1}\left[S\right]\left[E\right]+k_{2}\left[C\right]\\ \dot{\left[E\right]} & =-k_{1}\left[S\right]\left[E\right]+\left(k_{2}+k_{3}\right)\left[C\right]=-\dot{\left[C\right]}\\ \dot{\left[P\right]} & =k_{3}\left[C\right] \end{array} \end{array}$$ (24)

The enzyme model typically exhibits two time-scales: the binding and dissociation of *E* and *S* are usually much faster than the product formation, i.e., *k*_1_, *k*_2_ ≫ *k*_3_. This can lead to non-identifiable parameters *k*_1_ and *k*_2_. For large values of *k*_1_ and *k*_2_, the complex quickly reaches a quasi-equilibrium in which only the ratio of *k*_1_ and *k*_2_ can be determined.

**Fig 3:**
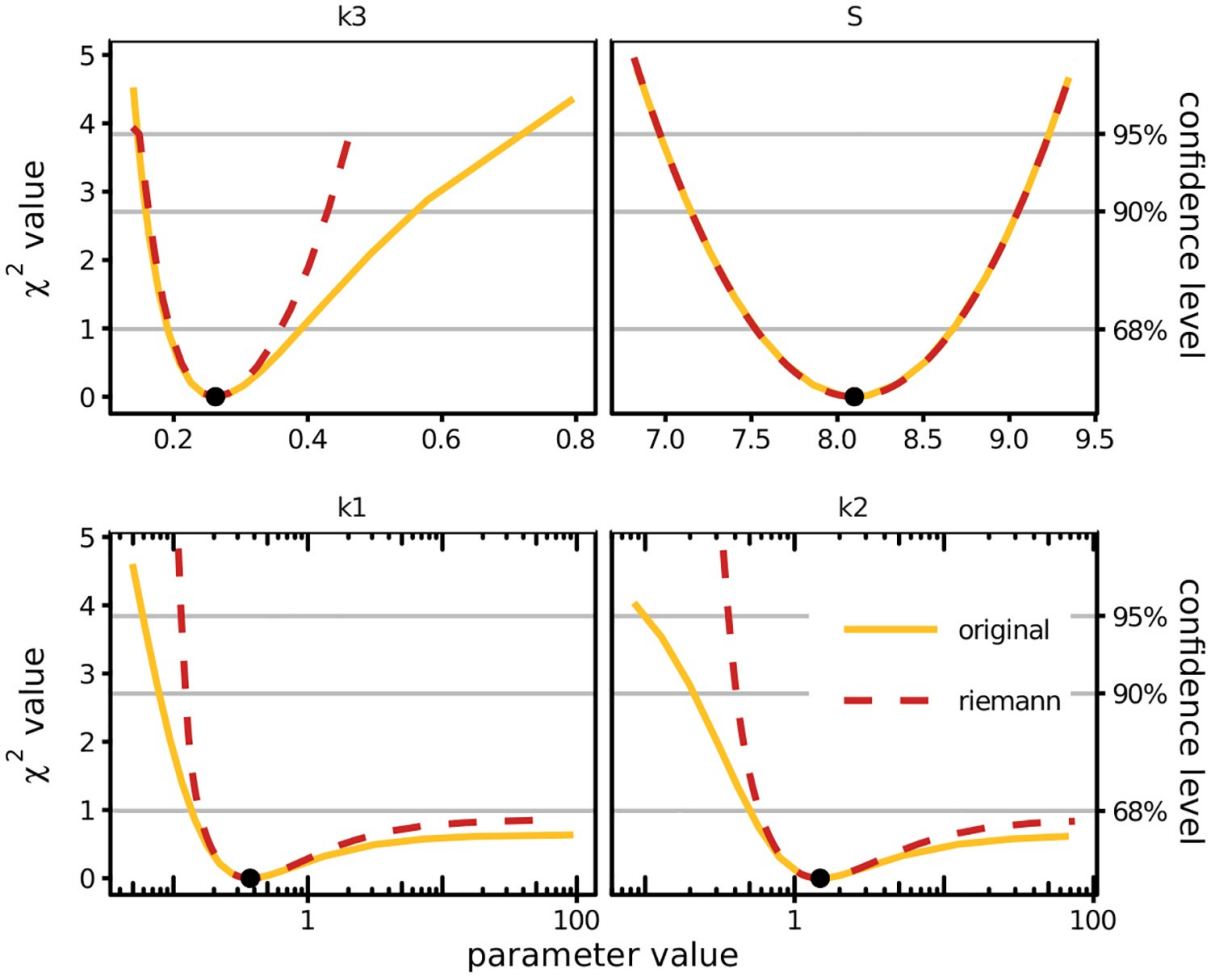
The profile likelihood for the parameter *k*_3_ and initial amount *S* indicates finite confidence intervals according to both, the original (yellow) and Riemann-approximated (red) *χ*^2^. The estimated parameter values are depicted as black dots. Practically non-identifiable parameters *k*_1_ and *k*_2_ do not even exceed the 68% confidence thresholds towards large values, correctly recognized by the approximation.

Because the visualization of higher-dimensional parameter spaces by contour lines is not feasible, we have evaluated the original and approximated objective function along profile-likelihood paths for different parameters. The simulated data is presented in section B in S1 Text. To identify coupled parameters, we again compare the profile likelihoods for *χ*^2^ and ${\tilde{\chi }}^{2}$. The production rate *k*_3_ and initial amount of substrate *S* are identifiable from the data as shown in Fig 3. For the parameters *S*, both the original and the approximated profiles are perfectly quadratic and coincide. The original profile of *k*_3_ is skewed. This skewness is visible in the approximation but it is not strong enough to reproduce the exact profile. As expected, the parameters *k*_1_ and *k*_2_ are practically non-identifiable. Again, the effect of practical non-identifiability is well captured by the approximating ${\tilde{\chi }}^{2}$, although not as strikingly as for the simpler model.

## Discussion

We established a connection between the Christoffel Symbols of a model and its boundedness. While models with globally vanishing Christoffel Symbols, i.e. linear models, are representatives of unbound models, models with constant Christoffel Symbols are bound in certain parameter directions and can therefore be considered as simple representatives of bounded models. We explored a possible application of this class of models as an approximation to non-linear least-squares, for which information about boundedness can be of importance in parameter uncertainty assessment. We now discuss various additional remarks concerning the quality, applicability and use of the approximation.

First, it should be noted that Christoffel Symbols depend on the coordinates from which they are calculated. The quality of the approximation by constant Christoffel Symbols therefore depends on the original coordinate system from which the coordinate change to the approximated Riemann Normal Coordinates is performed. If for example Model 1 was parameterized by $y=\frac{1}{\theta }$, *θ* > 0, the Christoffel Symbols would be given by $\Gamma_{11}^{1}=-\frac{2}{\theta }$. Thus, for different parameterizations of the same model manifold, the approximation of the Christoffel Symbols by constants yields different qualities of the respective approximations. For more complex models, in which the Christoffel Symbols cannot be written in closed form, the introduced errors may become difficult to quantify or to predict.

Furthermore, the parameterization of the model must not be redundant, such that the change of one parameter can exactly be compensated by another parameter. This setting is called “structural non-identifiability” [3] and in this case, the model manifold has a singular metric, which cannot be inverted. In this case, the structural non-identifiability has to be eliminated first, e.g. by methods described in [14].

Another issue is that the approximation by constant Christoffel Symbols could erroneously predict model manifold boundaries where there are none. This is exemplarily highlighted by the fact, that an unbound, one-dimensional polynomial model *y* = *θ*^*n*^, *n* being an uneven integer, has Christoffel Symbol $\Gamma_{11}^{1}=\frac{n-1}{\theta }$, a form which appears in the previous paragraph, associated with bounded manifolds. Therefore, the approach might be best suited to problems with a-priori known restricted model behaviors as in the presented examples. In this case, log-likelihood bounds will occur in certain directions of the parameter space. A question open to further research is if Christoffel Symbols obtained by second order sensitvities at the optimum are best suited to reproduce the models bounds for extreme parameter values or if the bounds could be reproduced better by other Christoffel Symbols. These could be obtained e.g. by sampling *χ*^2^ in the parameter space and might improve the distant approximation at the cost of reducing the quality of the local approximation close to the optimum.

On the statistical side regarding parameter identifiability, we explicitly note that while a model manifold or its approximation might be bounded in a certain region of parameter space, this does not necessarily imply that a parameter is practically non-identifiable, if the boundary is distant enough to the point of best fit such that the Δ*χ*^2^ threshold corresponding to a specific confidence level is crossed.

A possible application of the approximation is its use in parameter uncertainty assessment as done for Model 3. There are scenarios in which the approximation might be computationally cheaper than the original objective function. The original objective function might be much arbitrarily complex, whereas the structure of the approximated model is fixed to one evaluation of second order sensitivities of *χ*^2^ at $\hat{\theta }$ and, subsequently, to a two-point boundary value problem with *N* states. In practice, obtaining second order sensitivities can be challenging. For models formulated as ODEs, the numerically most stable way to obtain parameter derivatives is to integrate the sensitivity equations alongside the original ODEs [15]. Since the number of equations for second order sensitivity equations is $O(n^{3})$, already the algebraic derivation of these equation quickly becomes infeasible. Therefore, obtaining second order derivatives by finite differences might prove more viable in practice. Furthermore, the integration of the geodesic equation as a boundary value problem can be challenging and limits the approach to few parameters.

There are settings, though, in which the surrogate objective function ${\tilde{\chi }}^{2}$ could be evaluated faster than the original *χ*^2^. An example is given by an ODE model with many states, but few parameters. Such systems are frequent in rule-based biochemical models [16]. In this case, a complicated ODE with very many states but relatively few parameters could be replaced by a much simpler ODE with far fewer states. In a second scenario where the approximation might be beneficial, the data to be modeled consists of many experimental conditions, as is the case for dose-response experiments. This setting involves few unknown parameters as the stimulating concentrations in dose response experiments are usually fixed, but the ODE has to be evaluated many times with slightly different parameter values. In section C in S1 Text, we present runtimes and profile likelihood paths for a simulated dose-response experiment of Model 3 with 51 different enzyme concentrations. In this case, computation time could be saved compared to the original objective function.

## Conclusion

Parameter estimation in non-linear models is based on the optimization of an objective function, here denoted as *χ*^2^, which is non-quadratic, possibly non-convex, and features certain directions in which its values are bounded. This means that the position of the optimum in parameter space or the Hessian matrix around the optimum represent only a fraction of the information necessary to determine confidence intervals and relationships between model parameters in the case of practical non-identifiability.

In this work we have presented an approach based on differential geometry that, although using only second-order derivatives of the model at the optimum, provides an approximate *χ*^2^ that preserves the essential property of boundedness. Thereby, it allows to approximate *χ*^2^ of models with practically non-identifiable parameters surprisingly well and correctly predicts parameter coupling in the limit of infinitely large parameter values.

Despite this intriguing result, the local approximation of Christoffel Symbols bears also possible shortcomings. In our observation, the quality of the approximation decreases with increasing model size. Also the numerical solution of second-order sensitivity equations is limited by the mere number of equations. This raises the question if other, properly selected points in parameter space could be used to derive constant Christoffel Symbols.

In conclusion, the idea to capture the entire objective function of a non-linear model in a single matrix is tempting both from a conceptual and computational point of view. In the limit of many informative data points this idea is already realized by the quadratic form defined by the Hessian matrix around the optimum. In case of insufficient data we have shown that the geodesic equation with constant Christoffel Symbols can produce objective functions that approximate the original *χ*^2^ not only locally but also globally. Furthermore, for complex non-linear models with few parameters but high computational costs, the approximated objective function could be used to save computation time.

## Supporting information

S1 Text Details on methods and simulated data. (PDF)

S1 File Christoffel Symbols of Model 2 and 3, runtimes of the simulation experiments. (ZIP)

S2 File Computer code for simulation experiments. (ZIP)
