# Supplementary material for: Local Riemannian geometry of model manifolds and its implications for practical parameter identifiability
Source: PLoS One. 2019 Jun 3;14(6):e0217837. doi: 10.1371/journal.pone.0217837 (PMC6546239; doi:10.1371/journal.pone.0217837)
Supplement: S1 Text — (PDF) [file pone.0217837.s001.pdf]

SUPPLEMENTARY INFORMATION

A. Profile Likelihood

The profile likelihood is an effective method to find and explore canyons of χ^2 like the one in Figure 2 in the main text in a high dimensional parameter space. It is obtained by scanning through a single parameter θ^μ while re-optimizing likelihood function with respect to the other parameters [1]. For Gaussian noise and known variances, the log-likelihood function is just the sum of squares of the residuals plus a constant term. Therefore, the same profile paths are obtained by profiling χ^2 with respect to each parameter:

$$PL(\theta^\mu) = \min_{\nu \neq \mu} \chi^2(\theta^\nu) \tag{1}$$

To derive confidence intervals I_{θ^μ} from the profile likelihood, consider that PL specifies a submodel of the original model with the parameter θ^μ fixed to a certain value. These two models can now be compared by means of a likelihood ratio test with one degree of freedom. The submodel obtained by profile likelihood can be rejected for parameter values θ^μ at which PL is bigger than a threshold imposed by the desired confidence level. This leads to eq. 3 in the main text, which describes the confidence intervals obtained by profile likelihood.

$$I_{\theta^\mu} = \{\theta^\mu | PL(\theta^\mu) - \chi^2(\hat{\theta}) < T_{1-\alpha}\}, \tag{2}$$

where $T_{1-\alpha}$ is the threshold to be exceeded to guarantee a confidence level of $1-\alpha$. This threshold is derived from the inverse cumulative distribution function of a χ^2-distribution with one degree of freedom χ^2_1:

$$T_{1-\alpha} = icdf(\chi^2_1)(1-\alpha) \tag{3}$$

B. Description of the simulated data

We provide a description of the simulated data points which were used to generate Figure 2 and Figure 3 in the main text.

1. *Simulated data points for Figure 2*

The model of exponential decay, Model 1 in the main text, is specified as

$$\dot{A} = -k_1 A \quad \Leftrightarrow \quad A(t) = A_0 e^{-k_1 t}. \tag{4}$$

with two parameters, the initial amount $A_0 > 0$ and the decay constant $k_1 > 0$. The substance A is observed directly. Two data points are set as: $t_1 = 1, A_1 = 0.55, \sigma_1 = 0.04)$ and $(t_2 = 10, A_2 = 0.04, \sigma_2 = 0.04)$. In this case, the model manifold and data space have the same dimension. Consequently, the extrinsic curvature of the model manifold vanishes, eq. 11 becomes exact, and any difference between the original and the approximated χ^2 can be attributed to the zeroth order approximation of the Christoffel Symbols.

2. *Simulated data points for Figure 3*

name	time	value	sigma
enzyme	1.00	0.47	0.07
enzyme	300.00	0.32	0.07
substrate	1.00	8.15	1.46
substrate	75.75	2.94	0.66
substrate	150.50	1.78	0.24
substrate	225.25	0.27	0.07
substrate	300.00	0.09	0.02
product	1.00	0.05	0.01
product	75.75	3.73	0.82
product	150.50	10.46	1.24
product	225.25	7.52	1.41
product	300.00	5.11	1.46

TABLE I. Simulated data for Model 2 used to generate Figure 3 of the main text.

The ordinary differential equations of the model of enzyme kinetics, Model 2 in the main text, are given by

$$\begin{aligned} [\dot{S}] &= -k_1[S][E] + k_2[C] \\ [\dot{E}] &= -k_1[S][E] + (k_2 + k_3)[C] = -[\dot{C}] \\ [\dot{P}] &= k_3[C]. \end{aligned} \tag{5}$$

The observables to fully specify the model are given by:

$$\begin{aligned} y_1 &= E + C, && \text{(enzyme)} \\ y_2 &= S + C, && \text{(substrate)} \\ y_3 &= P. && \text{(product)} \end{aligned} \tag{6}$$

The initial concentrations of S and P are set to zero. The parameters k_1, k_2, k_3, and the remeaing initial concentrations E_0 and S_0 need to be estimated from the data. The simulated data is shown in Figure 1 as open squares with error bars and their numerical values are given in

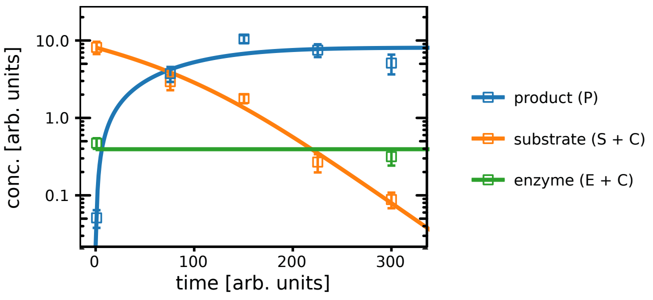

FIG. 1. Simulated data (dots and error bars) and model fit (solid lines) are shown.

Table I. The model fit obtained from the minimization of the corresponding χ^2 yields the solid lines shown in Figure 1. In this case, the number of data points exceeds the number of free parameters which suggests that the model manifold might be extrinsically curved. Therefore, larger deviations between original and approximated χ^2 are to be expected.

C. Comparison of runtimes

We compare the runtimes of the approximation for two different scenarios of data for Model 3, the enzyme kinetics example. The first scenario is data simulated similar to the data as presented in the previous section, SI, section B 2, whereas the second scenario is a simulated dose-response experiment with 50 different enzyme concentrations. Both simulation scenarios are provided in SI 3 as well as reproducible R-Scripts. The details of the data simulations are as follows.

- Each data point is simulated as $y_{D,m} = y(t_m, \theta_{\text{true}}) + \epsilon_m$, with the following "true" parameters:

$$
\begin{aligned}
S(0) &= 7.39 \text{ mol} \\
E(0) &= 1 \text{ mol} \\
C(0) &= 0 \text{ mol} \\
P(0) &= 0 \text{ mol} \\
k_1 &= 7.39 \text{ mol}^{-1}\text{s}^{-1} \\
k_2 &= 54.6 \text{ s}^{-1} \\
k_3 &= 0.36 \text{ s}^{-1}
\end{aligned} \tag{7}
$$

- As error model, we assume $\epsilon \sim \mathcal{N}(0, \sigma^2)$ with $\sigma = s0 + srel * y_D$. The parameters of the error model are given by $s0 = 0.05$ and $srel = 0.1$.
- The time points at which the data was taken are: $t_D = \{1s, 10s, 50s, 100s, 300s\}$
- The measured observables are the same as in equation (6)
- For the simulated dose-response experiment, 51 equidistant enzyme concentrations between 0 mol and 50 mol were used for used instead of the enzyme concentration given in (7). Furthermore, the data of the dose-response experiments were pooled and fitted simultaneously.

For the single-condition experiments, we simulated 100 data sets, for the dose-response experiments, 50 data sets were simulated. All parameters were log-transformed before the profile likelihood computations. Of the 100 single-dose data sets, for 24 data sets, the computation of the profiles for the RNC approximation failed due to a flat profile of either parameter log k_1 or log k_2, which indicates an ill-conditioned metric which causes numerical problems during the inversion necessary for the computation of the Christoffel Symbols.

The runtimes for the Profile Likelihood comutations are summarized by the Boxplot shown in Figure 2. Both original and RNC were computed with the same algorithm specifications for the profile likelihood computation to ensure positiveness of the parameters. It can be seen that for few data points, as in the single-condition experiment, the runtimes tend to vary strongly for RNC and that the original objective function is faster. However, for describing the dataset with many conditions, the original ODE describing the dynamics of the enzyme has to be integrated 51 times for one function evaluation. Furthermore, the number of steps to finish the profile likelihood computation up to the confidence level does not reach the user specified maximum, as is frequently the case for the data with one condition. Since the complexity of RNC has not increased in the dose-response setting, the approximation is faster in this case.

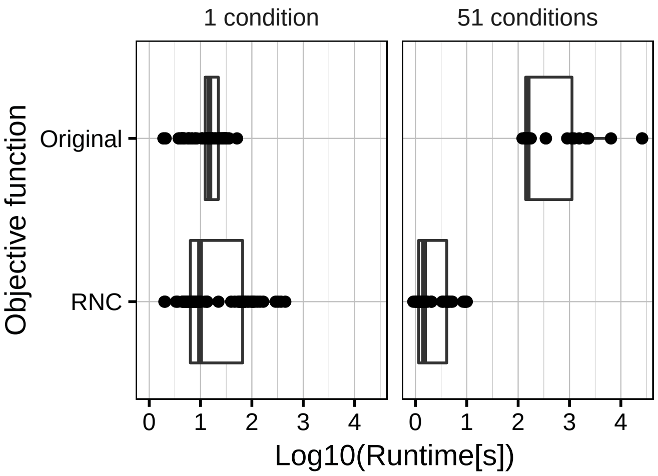

FIG. 2. Comparison of profile likelihood runtimes for the different objective function. **Left**: Scenario, in which the data has one condition only. Here, a great spread of runtimes is seen. **Right**: Data has 51 experimental conditions which require individual integration of the original ODE. Therefore, the runtimes of the original objective function are much higher in this setting. In contrast, the approximated objective function shows similar or even improved runtimes.

Regarding the quality of the approximation, we include exemplary figures of the profile likelihood paths. For the single-dose setting, in 24 cases, the approximation was not executable due to an ill-conditioned metric. In these cases, the computation of the Christoffel Symbols which requires an inversion of the metric led to numerical problems such that the approximation could not be computed at all. The most common shape of resulting profile likelihoods in these simulations, besides flat profiles of log k_1 and log k_2, can be seen in Figure

3. The profiles of the original objective function are depicted in black. The red curves show the approximation by constant Christoffel Symbols. The blue curves represent the quadratic approximation by the Fisher information matrix. Clearly, the practical non-identifiability for log $k_1 \to \infty$ and log $k_2 \to \infty$ is well captured, whereas the non-identifiability of log $k_2 \to -\infty$ cannot be reproduced. Furthermore, there appears to be a wrongly predicted practical non-identifiability of parameter log $k_3 \to -\infty$.

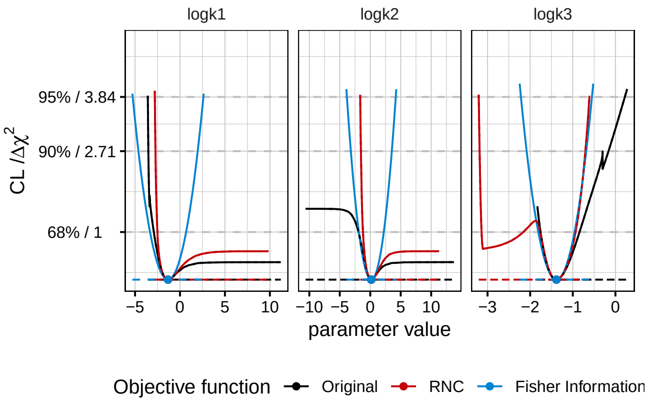

FIG. 3. Exemplary Profile Likelihood for the single-condition experiment. The R-Script "S1 Enzyme kinetics.R" of SI 3 was executed with random seed 17.

Interestingly, when more data is added, the approximation appears to improve. While the approximation in the single-dose setting only approximately finds the correct asympotic values, they are found with much greater reliability as the representative plot of profile likelihoods for the dose-response setting in Figure 4 shows.

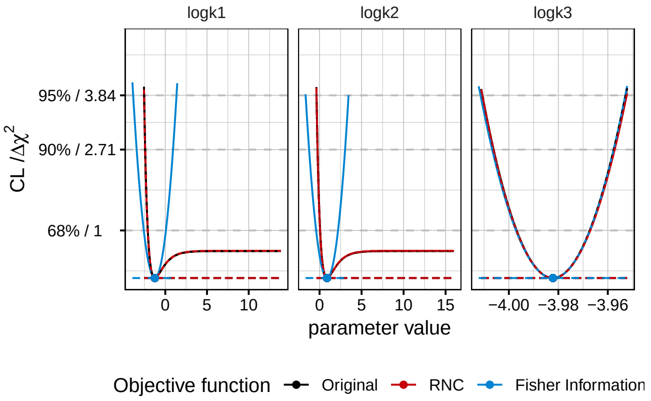

FIG. 4. Exemplary Profile Likelihood for the single-condition experiment. The R-Script "Script 1 Enzyme Kinetics Doseresponse.R" of SI 3 was executed with random seed 13.

D. Derivatives of the approximate objective function

Many algorithms dealing with objective functions also depend on derivatives of the same. Examples are given by Levenberg-Marquardt-like optimizers or integration-based profile likelihood calculation. In order to obtain derivatives of the new objective functions with respect to θ, it is possible to forward integrate the geodesic equation with sensitivity equations for the initial velocities. This yields the sensitivities $\frac{\partial \theta^\mu}{\partial v^\nu}$ which can be inverted to obtain $\frac{\partial v^\mu}{\partial \theta^{nu}}$ which are needed to obtain the gradient and an approximated Hessian matrix of $\tilde{\chi}^2$:

$$\begin{aligned} \frac{\partial(\hat{g}_{\mu\nu} v^\mu v^\nu)}{\partial \theta^\alpha} &= 2\hat{g}_{\mu\nu} v^\mu \frac{\partial v^\nu}{\partial \theta^\alpha} \\ \frac{\partial^2(\hat{g}_{\mu\nu} v^\mu v^\nu)}{\partial \theta^\alpha \partial \theta^\beta} &\approx 2\hat{g}_{\mu\nu} \frac{\partial v^\mu}{\partial \theta^\alpha} \frac{\partial v^\mu}{\partial \theta^\beta} \end{aligned} \tag{8}$$

E. Analytic solution of the geodesic equation for Model M1

Here, we present the steps which were used to arrive at eq. 18 in the main text.

$$\begin{aligned} \ddot{\theta} &= -(\Gamma^1_{11})\dot{\theta}\dot{\theta} = -(-1)\dot{\theta}^2 \\ \dot{\theta} &= -\frac{1}{\tau + \tilde{C}_0} \\ \theta(\tau) &= -\log(\tau + \tilde{C}_0) + \tilde{C}_1 \\ &= -\log(C_1\tau + C_0) \end{aligned} \tag{9}$$

In the last step, we changed the integration constants $\tilde{C}_i \to C_i$ such that their appearance fits our problem better.

Inserting the initial conditions $C_0 = 1$ and $C_1 = -v$, the geodesic is characterized by the following equations

$$\theta(\tau) = -\log(-v\tau + 1) \tag{10}$$

$$\dot{\theta} = v/(-v\tau + 1) \tag{11}$$

$$\theta(0) = -\log(1) \tag{12}$$

$$\theta(1) = -\log(-v + 1) \tag{13}$$

$$\dot{\theta}(0) = v \tag{14}$$

The domain of v is restricted by eq. (10) and is given by $\{v < 1 | v \in \mathbb{R}\}$.

[1] Raue, A., Kreutz, C., Maiwald, T., Bachmann, J., Schilling, M., Klingmüller, U., and Timmer, J. (2009). Structural and practical identifiability analysis of partially observed dynamical models by exploiting the profile likelihood. *Bioinformatics*, **25**(15), 1923–1929.
