# Supplementary material for: Local Riemannian geometry of model manifolds and its implications for practical parameter identifiability
Source: PLoS One. 2019 Jun 3;14(6):e0217837. doi: 10.1371/journal.pone.0217837 (PMC6546239; doi:10.1371/journal.pone.0217837)
Supplement: S2 File — (ZIP) [file pone.0217837.s003.zip › publication/packages/dMod2ndsens_0.2.1.tar.gz!/dMod2ndsens/README.html]

# dMod – Dynamic Modeling and Parameter Estimation R

The framework provides functions to generate ODEs of reaction networks, parameter transformations, observation functions, residual functions, etc. The framework follows the paradigm that derivative information should be used for optimization whenever possible. Therefore, all major functions produce and can handle expressions for symbolic derivatives.

## Simple example: enzyme kinetics

### Load required packages

```
library(deSolve)
library(dMod)
```

### Generate an ODE model of enzyme kinetics with enzyme degradation

```
f <- eqnlist()
f <- addReaction(f, from = "Enz + Sub", to = "Compl", rate = c("production of complex" = "k1*Enz*Sub"))
f <- addReaction(f, from = "Compl", to = "Enz + Sub", rate = c("decay of complex" = "k2*Compl"))
f <- addReaction(f, from = "Compl", to = "Enz + Prod", rate = c("production of product" = "k3*Compl"))
f <- addReaction(f, from = "Enz", to = ""     , rate = c("enzyme degradation" = "k4*Enz"))
eqns <- as.eqnvec(f)
model <- generateModel(eqns, modelname = "enzymeKinetics")
```

### Define observables and generate observation function `g`

```
observables <- eqnvec(c(product = "Prod", substrate = "(Sub + Compl)", enzyme = "(Enz + Compl)"))

# Generate observation functions2
g <- Y(observables, eqns, compile = TRUE, modelname = "obsfn")
```

### Define parameter transformation for two experimental conditions

```
# Get all parameters
innerpars <- getSymbols(c(eqns, names(eqns), observables))
# Symbolically write down a log-transform
trafo1 <- trafo2 <- structure(paste0("exp(log", innerpars, ")"), names = innerpars)
# Set some initial parameters
trafo1["Compl"] <- trafo2["Compl"] <- "0"
trafo1["Prod"] <- trafo2["Prod"] <- "0"
# Set the degradation rate in the first condition to 0
trafo1["k4"] <- "0"
# Get names of the new parameters
outerpars <- getSymbols(c(trafo1, trafo2))

# Generate parameter transformation functions


pL <- list(noDegradation = P(trafo1), 
           withDegradation = P(trafo2))

conditions <- names(pL)

# Initialize with randomly chosen parameters
set.seed(1)
pouter <- structure(rnorm(length(outerpars), -2, .5), names = outerpars)
```

### Define the model prediction function

```
# Generate low-level prediction function
x0 <- Xs(model$func, model$extended)

# Generate a high-level prediction function: trafo -> prediction -> observation
x <- prdfn({
  pinner <- pL[[condition]](pars, fixed)
  prediction <- x0(times, pinner, ...)
  g(prediction, pinner, attach.input = TRUE)
}, pouter = pouter, conditions = conditions)


times <- 0:100

plot(x(times, pouter))
```

### Define data to be fitted by the model

```
data <- datalist(
  list(
    noDegradation = data.frame(
      name = c("product", "product", "product", "substrate", "substrate", "substrate"),
      time = c(0, 25, 100, 0, 25, 100),
      value = c(0.0025, 0.2012, 0.3080, 0.3372, 0.1662, 0.0166),
      sigma = 0.02),
    withDegradation = data.frame(
      name = c("product", "product", "product", "substrate", "substrate", "substrate", "enzyme", "enzyme", "enzyme"),
      time = c(0, 25, 100, 0, 25, 100, 0, 25, 100),
      value = c(-0.0301,  0.1512, 0.2403, 0.3013, 0.1635, 0.0411, 0.4701, 0.2001, 0.0383),
      sigma = 0.02)
  )
)

timesD <- sort(unique(unlist(lapply(data, function(d) d$time))))

# Compare data to prediction
plot(data) + geom_line()
```

```
plot(x(times, pouter), data)
```

### Define an objective function to be minimized and run minimization by `trust()`

```
obj <- objfn(data = data, x = x, pouter = pouter, conditions = conditions, {
  constraintL2(pouter, prior, sigma = 10)
})

# Optimize the objective function
prior <- structure(rep(0, length(pouter)), names = names(pouter))
myfit <- trust(obj, pouter, rinit = 1, rmax = 10)

plot(x(times, myfit$argument), data)
```

### Compute the profile likelihood to analyze parameter identifiability

```
library(parallel)
bestfit <- myfit$argument
profiles <- mclapply(names(bestfit), function(n) profile(obj, bestfit, n, limits=c(-10, 10)), mc.cores=4)
names(profiles) <- names(bestfit)

# Take a look at each parameter
plotProfile(profiles)
```

```
# Compare parameters and their confidence intervals
plotProfile(profiles) + facet_wrap(~name, ncol = 1)
```
