# Supplementary material for: Local Riemannian geometry of model manifolds and its implications for practical parameter identifiability
Source: PLoS One. 2019 Jun 3;14(6):e0217837. doi: 10.1371/journal.pone.0217837 (PMC6546239; doi:10.1371/journal.pone.0217837)
Supplement: S2 File — (ZIP) [file pone.0217837.s003.zip › publication/packages/cOde2ndSens_0.2.tar.gz!/cOde2ndSens/README.html]

Automated C Code Generation for Use with the deSolve and bvpSolve Packages


# Automated C Code Generation for Use with the ‘deSolve’ and ‘bvpSolve’ Packages

#### *Daniel Kaschek*

#### *September 29, 2015*

cOde generates all necessary C functions allowing the user to work with the compiled-code interface of ode() and bvptwp(). The implementation supports “forcings” and “events”. The package also provides functions to symbolically compute Jacobians, sensitivity equations and adjoint sensitivities being the basis for sensitivity analysis.

## First example: exponential decay

\[A \stackrel{k1}{\rightarrow} B \stackrel{k2}{\rightarrow} \emptyset\]

```
library(cOde)
library(deSolve)

# Define ODE as character vector
f <- c(
  A = "-k1*A",
  B = "k1*A - k2*B"
)

# Auto-generate C code and compile it
func <- funC(f)

# Integrate (compiled) ODE by wrapper function odeC
times <- 0:100
yini <- c(A = 1, B = 0)
parms <- c(k1 = 2e-1, k2 = 1e-1)

out <- odeC(y = yini, times = times, func = func, parms = parms)

# Plot the result
with(as.data.frame(out), matplot(x = time, y = cbind(A, B), type = "l", lty = 1))
```

## Second example: sensitivity equations

Given the ODE \(\dot x = f(x, p)\), generate the sensitivity equations and integrate the together with the original ODE.

```
# Compute sensitivity equations of f
f.sens <- sensitivitiesSymb(f)

# Auto-generate C code of the combined ODE system and compile it.
func <- funC(c(f, f.sens))

# Solve the ODE by lsodes()
yini <- c(yini, attr(f.sens, "yini"))
out <- odeC(y = yini, times = times, func = func, parms = parms, method = "lsodes")

# Plot the result (plot shows the sensitivities of B)
with(as.data.frame(out), matplot(x = time, y = cbind(B.k1, B.k2, B.A, B.B), type = "l", lty = 1))
```
